# 3-Oxoolean-1-en-28-oic acid–*n*-hexa­ne–water (4/1/1) from the bark of *Walsura pinnata* Hassk

**DOI:** 10.1107/S1600536809015086

**Published:** 2009-04-30

**Authors:** Khalijah Awang, Mahfizah Yusoff, Khalit Mohamad, Soo Lim Chong, Seik Weng Ng

**Affiliations:** a Department of Chemistry, University of Malaya, 50603 Kuala Lumpur, Malaysia

## Abstract

3-Oxoolean-1-en-28-oic acid, isolated from the bark of *Walsura pinnata* Hassk, crystallized from *n*-hexane as an *n*-hexane 0.25-solvent 0.25-hydrate, C_30_H_46_O_3_·0.25C_6_H_14_·0.25H_2_O. There are two independent mol­ecules in the asymmetric unit of the title compound. The three six-membered cyclo­hexane rings in each mol­ecule adopt chair conformations and the carboxyl substituent occupies an axial/equatorial position. The two independent mol­ecules are linked by a pair of O—H_carbox­yl_⋯O hydrogen bonds into a dimer. The *n*-hexane mol­ecule is disordered about a twofold rotation axis and the water mol­ecule lies on a twofold rotation axis. In addition, the cyclo­hexone carbonyl group of one of the independent mol­ecules is disordered over two sites with occupancies of 0.75 and 0.25.

## Related literature

There are no reports of chemicals from *Walsura pinnata* Hassk. For the action of a fungus on this compound, isolated from another source, see: Shirane *et al.* (1996 ▶).
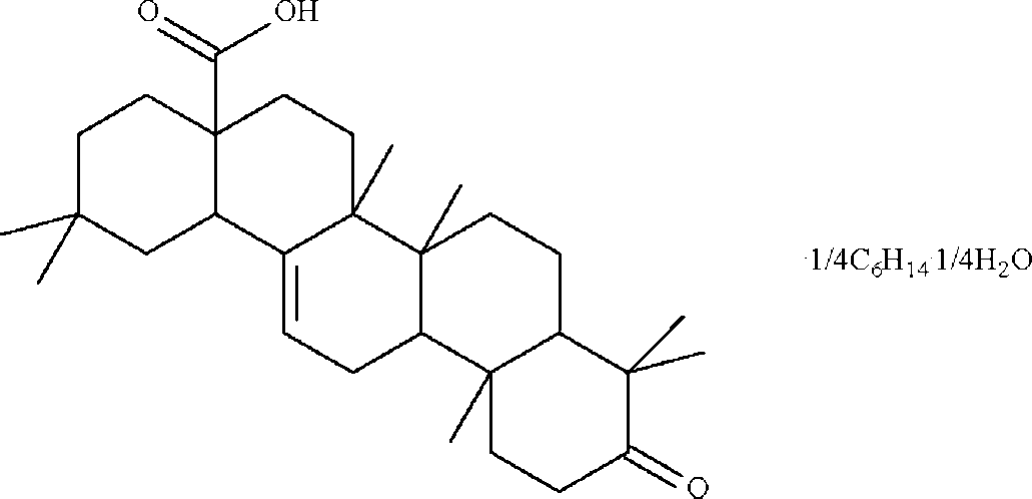


## Experimental

### 

#### Crystal data

C_30_H_46_O_3_·0.25C_6_H_14_·0.25H_2_O
*M* *_r_* = 480.71
Monoclinic, 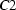

*a* = 28.5864 (7) Å
*b* = 12.2408 (3) Å
*c* = 19.3545 (4) Å
β = 120.552 (1)°
*V* = 5832.3 (2) Å^3^
*Z* = 8
Mo *K*α radiation
μ = 0.07 mm^−1^
*T* = 100 K
0.45 × 0.15 × 0.10 mm

#### Data collection

Bruker SMART APEX diffractometer
Absorption correction: none
20554 measured reflections
6997 independent reflections
5441 reflections with *I* > 2σ(*I*)
*R*_int_ = 0.038

#### Refinement

*R*[*F* ^2^ > 2σ(*F* ^2^)] = 0.068
*wR*(*F* ^2^) = 0.204
*S* = 1.07
6997 reflections
676 parameters
71 restraints
H-atom parameters constrained
Δρ_max_ = 0.81 e Å^−3^
Δρ_min_ = −0.62 e Å^−3^

### 

Data collection: *APEX2* (Bruker, 2007 ▶); cell refinement: *SAINT* (Bruker, 2007 ▶); data reduction: *SAINT*; program(s) used to solve structure: *SHELXS97* (Sheldrick, 2008 ▶); program(s) used to refine structure: *SHELXL97* (Sheldrick, 2008 ▶); molecular graphics: *X-SEED* (Barbour, 2001 ▶); software used to prepare material for publication: *publCIF* (Westrip, 2009 ▶).

## Supplementary Material

Crystal structure: contains datablocks I, global. DOI: 10.1107/S1600536809015086/lh2809sup1.cif

Structure factors: contains datablocks I. DOI: 10.1107/S1600536809015086/lh2809Isup2.hkl

Additional supplementary materials:  crystallographic information; 3D view; checkCIF report

## Figures and Tables

**Table 1:** Hydrogen-bond geometry (Å, °)

*D*—H⋯*A*	*D*—H	H⋯*A*	*D*⋯*A*	*D*—H⋯*A*
O1—H1⋯O4	0.84	1.71	2.545 (4)	171
O5—H5⋯O2	0.84	1.83	2.637 (4)	160
O1w—H1w⋯O3	0.84	2.20	3.03 (2)	169

**Fig. 1.**
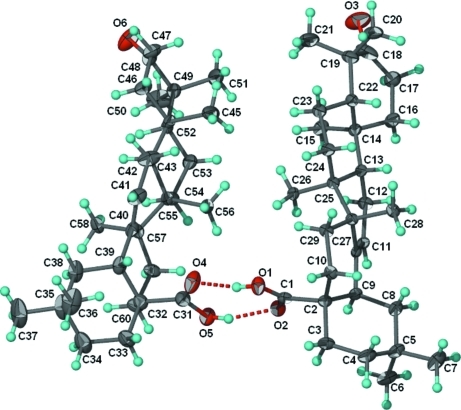
70% Probability thermal-ellipsoid plot (Barbour, 2001) of the two independent molecules of C30H46O3.1/4C6H14.1/4H2O. Hydrogen atoms are drawn as spheres of arbitrary radii. The disorder in one of the cyclohexanone carbonyl groups and the solvent molecules are not shown.

## supplementary crystallographic information

### Comment

The two independent molecules are shown in Fig. 1.

### Experimental

The dried and ground bark of *Walsura pinnata* Hassk (2.3 kg) was extracted with *n*-hexane for 72 h at room temperature. The solvent was evaporated to give a crude extract, which was subjected to column chromatography on silica gel (60 GF_254_) by using *n*-hexane with increasing amounts of ethyl acetate as eluent. Of the twenty-four fractions collected, the twenty-second fraction, eluted with ethyl acetate:*n*-hexane, 14:86 gave 2 g of the product, which was further purified by column chromatography (*n*-hexane:ethyl acetate 80:20) to give the title compound (10 mg).

The formulation was established by satisfactory solution NMR spectroscopy.

### Refinement

The carbonyl group of one of the two independent molecules is disordered over two positions. For this unit – C17–C18(=O3)–C19 – the 1,2- and 1,3-related distances of the umprimed and primed atoms were restrained to within 0.01 Å of each other. The temperature factors of the umprimed atoms were set to those of the prime ones for the C18/C18' and O3/O3' atoms. The four-atom unit was restrained to be nearly planar.

The hexane molecule lies on a twofold rotation axis; the molecule was instead refined as a six-carbon species, with 1,2- and 1,3-related distances being restrained to 1.54±0.01 and 2.51±0.01 Å. The anisotropic temperature factors of the six carbon atoms were restrained to be nearly isotropic.

The water molecules lies on a twofold rotation axis; the oxygen atom showed large anisotropic temperature factors.

Carbon-bound H-atoms were placed in calculated positions (C—H 0.95–0.99 Å) and were included in the refinement in the riding model approximation, with *U*(H) set to 1.2–1.5*U*_eq_(C). The acid H-atoms were placed in chemically sensible positions but were not refined.

In the absence of heavy scatterers, Friedel pairs were merged.

### Crystal data

**Table:**

C_30_H_46_O_3_·0.25C_6_H_14_·0.25H_2_O	*F*(000) = 2120
*M**_r_* = 480.71	*D*_x_ = 1.095 Mg m^−^^3^
Monoclinic, *C*2	Mo *K*α radiation, λ = 0.71073 Å
Hall symbol: C 2y	Cell parameters from 4238 reflections
*a* = 28.5864 (7) Å	θ = 2.4–22.7°
*b* = 12.2408 (3) Å	µ = 0.07 mm^−^^1^
*c* = 19.3545 (4) Å	*T* = 100 K
β = 120.552 (1)°	Colorless, prism
*V* = 5832.3 (2) Å^3^	0.45 × 0.15 × 0.10 mm
*Z* = 8	

### Data collection

**Table:**

Bruker SMART APEX diffractometer	5441 reflections with *I* > 2σ(*I*)
Radiation source: fine-focus sealed tube	*R*_int_ = 0.038
graphite	θ_max_ = 27.5°, θ_min_ = 1.2°
ω scans	*h* = −37→36
20554 measured reflections	*k* = −15→15
6997 independent reflections	*l* = −25→24

### Refinement

**Table:**

Refinement on *F*^2^	Primary atom site location: structure-invariant direct methods
Least-squares matrix: full	Secondary atom site location: difference Fourier map
*R*[*F*^2^ > 2σ(*F*^2^)] = 0.068	Hydrogen site location: inferred from neighbouring sites
*wR*(*F*^2^) = 0.204	H-atom parameters constrained
*S* = 1.07	*w* = 1/[σ^2^(*F*_o_^2^) + (0.1276*P*)^2^ + 2.2067*P*] where *P* = (*F*_o_^2^ + 2*F*_c_^2^)/3
6997 reflections	(Δ/σ)_max_ = 0.001
676 parameters	Δρ_max_ = 0.81 e Å^−^^3^
71 restraints	Δρ_min_ = −0.62 e Å^−^^3^

### Fractional atomic coordinates and isotropic or equivalent isotropic displacement parameters (Å^2^)

**Table:**

	*x*	*y*	*z*	*U*_iso_*/*U*_eq_	Occ. (<1)
O1	0.64169 (14)	0.4999 (3)	0.06365 (17)	0.0372 (7)	
H1	0.6626	0.5079	0.1133	0.056*	
O2	0.64271 (12)	0.6807 (3)	0.06809 (17)	0.0338 (7)	
O3	0.9328 (2)	0.6613 (5)	−0.1228 (4)	0.0706 (16)	0.75
O3'	0.8798 (6)	0.6340 (10)	−0.2241 (7)	0.0706 (16)	0.25
O4	0.71245 (16)	0.5134 (3)	0.21228 (19)	0.0543 (10)	
O5	0.70076 (14)	0.6881 (3)	0.22605 (19)	0.0427 (8)	
H5	0.6763	0.6814	0.1777	0.064*	
O6	1.12997 (15)	0.6700 (4)	0.3873 (3)	0.0672 (12)	
O1W	1.0000	0.834 (2)	0.0000	0.248 (9)	
H1W	0.9789	0.7934	−0.0385	0.371*	
C1	0.62647 (15)	0.5947 (4)	0.0301 (2)	0.0268 (8)	
C2	0.58412 (15)	0.5936 (4)	−0.0588 (2)	0.0258 (8)	
C3	0.52936 (16)	0.5977 (4)	−0.0593 (3)	0.0338 (9)	
H3A	0.5240	0.5273	−0.0391	0.041*	
H3B	0.5314	0.6560	−0.0224	0.041*	
C4	0.48058 (17)	0.6192 (4)	−0.1430 (3)	0.0399 (11)	
H4A	0.4758	0.5562	−0.1781	0.048*	
H4B	0.4474	0.6253	−0.1392	0.048*	
C5	0.48699 (17)	0.7227 (4)	−0.1809 (3)	0.0371 (10)	
C6	0.4882 (2)	0.8240 (5)	−0.1331 (3)	0.0462 (12)	
H6A	0.4548	0.8267	−0.1305	0.069*	
H6B	0.4908	0.8899	−0.1596	0.069*	
H6C	0.5197	0.8198	−0.0786	0.069*	
C7	0.43893 (19)	0.7325 (5)	−0.2667 (3)	0.0505 (13)	
H7A	0.4051	0.7373	−0.2657	0.076*	
H7B	0.4377	0.6681	−0.2977	0.076*	
H7C	0.4432	0.7984	−0.2918	0.076*	
C8	0.54003 (16)	0.7146 (4)	−0.1828 (2)	0.0308 (9)	
H8A	0.5450	0.7833	−0.2054	0.037*	
H8B	0.5364	0.6544	−0.2193	0.037*	
C9	0.59140 (15)	0.6944 (3)	−0.1001 (2)	0.0252 (8)	
H9	0.5960	0.7592	−0.0657	0.030*	
C10	0.64277 (14)	0.6855 (3)	−0.1054 (2)	0.0213 (7)	
C11	0.67132 (16)	0.7747 (3)	−0.0975 (2)	0.0247 (8)	
H11	0.6585	0.8412	−0.0878	0.030*	
C12	0.72209 (16)	0.7808 (3)	−0.1023 (2)	0.0265 (8)	
H12A	0.7522	0.8074	−0.0503	0.032*	
H12B	0.7165	0.8349	−0.1439	0.032*	
C13	0.73895 (15)	0.6714 (3)	−0.1223 (2)	0.0215 (7)	
H13	0.7143	0.6629	−0.1815	0.026*	
C14	0.79755 (15)	0.6718 (3)	−0.1098 (2)	0.0248 (8)	
C15	0.84201 (17)	0.7066 (4)	−0.0258 (3)	0.0335 (9)	
H15A	0.8762	0.7167	−0.0252	0.050*	
H15B	0.8467	0.6500	0.0130	0.050*	
H15C	0.8316	0.7755	−0.0115	0.050*	
C16	0.79644 (18)	0.7532 (4)	−0.1717 (3)	0.0363 (10)	
H16A	0.7663	0.7334	−0.2258	0.044*	
H16B	0.7892	0.8274	−0.1591	0.044*	
C17	0.8498 (2)	0.7548 (4)	−0.1727 (3)	0.0431 (12)	
H17A	0.8412	0.7684	−0.2284	0.052*	
H17B	0.8722	0.8166	−0.1394	0.052*	
C18	0.8821 (3)	0.6541 (6)	−0.1435 (3)	0.0526 (19)	0.75
C18'	0.8654 (3)	0.6426 (8)	−0.1818 (6)	0.0526 (19)	0.25
C19	0.86193 (19)	0.5453 (4)	−0.1348 (3)	0.0376 (10)	
C20	0.8513 (2)	0.4719 (6)	−0.2056 (3)	0.0541 (15)	
H20A	0.8854	0.4606	−0.2053	0.081*	
H20B	0.8247	0.5070	−0.2560	0.081*	
H20C	0.8371	0.4013	−0.2007	0.081*	
C21	0.9086 (2)	0.4917 (8)	−0.0581 (3)	0.070 (2)	
H21A	0.9407	0.4844	−0.0636	0.104*	
H21B	0.8972	0.4193	−0.0507	0.104*	
H21C	0.9175	0.5376	−0.0115	0.104*	
C22	0.80785 (16)	0.5581 (3)	−0.1335 (2)	0.0257 (8)	
H22	0.7780	0.5468	−0.1902	0.031*	
C23	0.80078 (17)	0.4670 (3)	−0.0851 (2)	0.0291 (8)	
H23A	0.8098	0.3956	−0.0993	0.035*	
H23B	0.8258	0.4795	−0.0270	0.035*	
C24	0.74204 (17)	0.4658 (3)	−0.1034 (2)	0.0273 (8)	
H24A	0.7381	0.4075	−0.0712	0.033*	
H24B	0.7177	0.4476	−0.1607	0.033*	
C25	0.72373 (15)	0.5751 (3)	−0.0854 (2)	0.0225 (7)	
C26	0.75144 (15)	0.5890 (4)	0.0067 (2)	0.0259 (8)	
H26A	0.7907	0.5791	0.0312	0.039*	
H26B	0.7371	0.5343	0.0281	0.039*	
H26C	0.7440	0.6623	0.0190	0.039*	
C27	0.65940 (15)	0.5761 (3)	−0.1251 (2)	0.0225 (7)	
C28	0.62911 (16)	0.5670 (3)	−0.2184 (2)	0.0275 (8)	
H28A	0.5908	0.5494	−0.2389	0.041*	
H28B	0.6458	0.5091	−0.2338	0.041*	
H28C	0.6317	0.6366	−0.2412	0.041*	
C29	0.64230 (16)	0.4771 (3)	−0.0940 (2)	0.0251 (8)	
H29A	0.6412	0.4117	−0.1248	0.030*	
H29B	0.6704	0.4643	−0.0372	0.030*	
C30	0.58735 (16)	0.4889 (3)	−0.0997 (2)	0.0259 (8)	
H30A	0.5811	0.4250	−0.0741	0.031*	
H30B	0.5583	0.4900	−0.1569	0.031*	
C31	0.72161 (17)	0.5932 (4)	0.2551 (2)	0.0339 (9)	
C32	0.75531 (17)	0.5834 (4)	0.3459 (2)	0.0339 (9)	
C33	0.7125 (2)	0.5534 (5)	0.3692 (3)	0.0433 (11)	
H33A	0.6899	0.6185	0.3623	0.052*	
H33B	0.6883	0.4959	0.3324	0.052*	
C34	0.7382 (2)	0.5129 (6)	0.4555 (3)	0.0551 (15)	
H34A	0.7090	0.4893	0.4655	0.066*	
H34B	0.7578	0.5741	0.4924	0.066*	
C35	0.7776 (2)	0.4181 (6)	0.4742 (3)	0.0597 (17)	
C36	0.7479 (3)	0.3152 (6)	0.4269 (4)	0.071 (2)	
H36A	0.7736	0.2542	0.4438	0.106*	
H36B	0.7326	0.3285	0.3694	0.106*	
H36C	0.7186	0.2972	0.4372	0.106*	
C37	0.8052 (3)	0.3955 (8)	0.5653 (4)	0.084 (3)	
H37A	0.8302	0.3336	0.5795	0.126*	
H37B	0.7775	0.3782	0.5791	0.126*	
H37C	0.8255	0.4604	0.5952	0.126*	
C38	0.8213 (2)	0.4554 (5)	0.4546 (3)	0.0468 (13)	
H38A	0.8422	0.5163	0.4907	0.056*	
H38B	0.8468	0.3942	0.4654	0.056*	
C39	0.79764 (18)	0.4926 (4)	0.3673 (2)	0.0359 (10)	
H39	0.7778	0.4285	0.3327	0.043*	
C40	0.84081 (17)	0.5249 (4)	0.3471 (2)	0.0314 (9)	
C41	0.85843 (19)	0.4506 (4)	0.3156 (3)	0.0413 (11)	
H41	0.8428	0.3798	0.3068	0.050*	
C42	0.9010 (2)	0.4683 (5)	0.2927 (3)	0.0489 (13)	
H42A	0.8839	0.4606	0.2338	0.059*	
H42B	0.9290	0.4105	0.3179	0.059*	
C43	0.92871 (16)	0.5796 (4)	0.3176 (2)	0.0305 (9)	
H43	0.9536	0.5741	0.3770	0.037*	
C44	0.96696 (16)	0.6060 (4)	0.2835 (2)	0.0329 (10)	
C45	0.9375 (2)	0.6018 (6)	0.1903 (3)	0.0561 (16)	
H45A	0.9182	0.6707	0.1680	0.084*	
H45B	0.9114	0.5413	0.1706	0.084*	
H45C	0.9643	0.5906	0.1736	0.084*	
C46	1.01230 (18)	0.5192 (4)	0.3172 (3)	0.0396 (11)	
H46A	1.0265	0.5103	0.3754	0.048*	
H46B	0.9969	0.4483	0.2908	0.048*	
C47	1.0592 (2)	0.5498 (5)	0.3042 (3)	0.0451 (12)	
H47A	1.0879	0.4935	0.3293	0.054*	
H47B	1.0459	0.5506	0.2460	0.054*	
C48	1.08275 (19)	0.6585 (5)	0.3391 (3)	0.0419 (11)	
C49	1.0432 (2)	0.7552 (4)	0.3119 (3)	0.0423 (11)	
C50	1.0716 (3)	0.8501 (5)	0.3670 (5)	0.0698 (19)	
H50A	1.0748	0.8366	0.4191	0.105*	
H50B	1.0505	0.9170	0.3435	0.105*	
H50C	1.1079	0.8586	0.3742	0.105*	
C51	1.0297 (3)	0.7881 (7)	0.2266 (4)	0.073 (2)	
H51A	1.0634	0.8057	0.2273	0.109*	
H51B	1.0059	0.8522	0.2089	0.109*	
H51C	1.0114	0.7273	0.1896	0.109*	
C52	0.99266 (18)	0.7183 (4)	0.3177 (2)	0.0339 (10)	
H52	1.0073	0.7109	0.3766	0.041*	
C53	0.9495 (2)	0.8059 (4)	0.2916 (3)	0.0425 (12)	
H53A	0.9273	0.8078	0.2323	0.051*	
H53B	0.9670	0.8782	0.3104	0.051*	
C54	0.9129 (2)	0.7824 (4)	0.3265 (3)	0.0370 (10)	
H54A	0.8839	0.8383	0.3062	0.044*	
H54B	0.9348	0.7901	0.3855	0.044*	
C55	0.88640 (17)	0.6687 (4)	0.3064 (2)	0.0302 (9)	
C56	0.83882 (18)	0.6701 (5)	0.2192 (2)	0.0452 (13)	
H56A	0.8526	0.6849	0.1830	0.068*	
H56B	0.8130	0.7274	0.2130	0.068*	
H56C	0.8205	0.5991	0.2060	0.068*	
C57	0.86480 (17)	0.6396 (4)	0.3657 (2)	0.0298 (9)	
C58	0.91077 (17)	0.6397 (4)	0.4546 (2)	0.0330 (9)	
H58A	0.8949	0.6390	0.4891	0.050*	
H58B	0.9331	0.7054	0.4656	0.050*	
H58C	0.9335	0.5747	0.4654	0.050*	
C59	0.82326 (18)	0.7272 (4)	0.3573 (3)	0.0332 (9)	
H59A	0.8433	0.7924	0.3887	0.040*	
H59B	0.8016	0.7494	0.3003	0.040*	
C60	0.78427 (18)	0.6904 (4)	0.3852 (3)	0.0359 (10)	
H60A	0.8049	0.6809	0.4442	0.043*	
H60B	0.7568	0.7482	0.3724	0.043*	
C61	0.8146 (6)	1.0554 (15)	0.3756 (8)	0.092 (5)	0.50
H61A	0.7983	0.9941	0.3382	0.138*	0.50
H61B	0.8207	1.1164	0.3483	0.138*	0.50
H61C	0.7900	1.0786	0.3941	0.138*	0.50
C63	0.8949 (7)	1.1153 (17)	0.5058 (10)	0.139 (8)	0.50
H63A	0.9253	1.0880	0.5573	0.167*	0.50
H63B	0.8683	1.1517	0.5163	0.167*	0.50
C64	0.9164 (11)	1.197 (2)	0.4664 (11)	0.179 (12)	0.50
H64A	0.9315	1.1571	0.4376	0.215*	0.50
H64B	0.8866	1.2449	0.4277	0.215*	0.50
C62	0.8679 (7)	1.0202 (13)	0.4464 (10)	0.128 (7)	0.50
H62A	0.8924	0.9947	0.4275	0.153*	0.50
H62B	0.8618	0.9585	0.4739	0.153*	0.50
C65	0.9612 (9)	1.2658 (19)	0.5355 (12)	0.142 (9)	0.50
H65A	0.9458	1.3053	0.5640	0.170*	0.50
H65B	0.9905	1.2172	0.5743	0.170*	0.50
C66	0.9844 (11)	1.347 (2)	0.5009 (18)	0.160 (12)	0.50
H66A	1.0132	1.3901	0.5446	0.240*	0.50
H66B	0.9554	1.3966	0.4636	0.240*	0.50
H66C	0.9992	1.3079	0.4723	0.240*	0.50

### Atomic displacement parameters (Å^2^)

**Table:**

	*U*^11^	*U*^22^	*U*^33^	*U*^12^	*U*^13^	*U*^23^
O1	0.0479 (19)	0.0291 (17)	0.0274 (14)	0.0008 (14)	0.0138 (14)	−0.0006 (13)
O2	0.0416 (17)	0.0257 (15)	0.0323 (14)	0.0010 (13)	0.0173 (13)	−0.0036 (13)
O3	0.050 (3)	0.062 (3)	0.116 (4)	0.006 (3)	0.054 (3)	0.021 (3)
O3'	0.050 (3)	0.062 (3)	0.116 (4)	0.006 (3)	0.054 (3)	0.021 (3)
O4	0.064 (2)	0.038 (2)	0.0313 (16)	0.0119 (18)	0.0029 (16)	−0.0100 (15)
O5	0.049 (2)	0.0372 (19)	0.0327 (15)	0.0086 (16)	0.0144 (14)	−0.0053 (14)
O6	0.037 (2)	0.050 (2)	0.083 (3)	−0.0008 (18)	0.0078 (19)	0.010 (2)
O1W	0.249 (12)	0.228 (13)	0.248 (12)	0.000	0.113 (9)	0.000
C1	0.0260 (18)	0.027 (2)	0.0327 (18)	0.0002 (17)	0.0190 (16)	−0.0016 (18)
C2	0.0213 (17)	0.028 (2)	0.0282 (17)	−0.0006 (16)	0.0130 (14)	−0.0018 (17)
C3	0.0272 (19)	0.039 (2)	0.041 (2)	−0.0017 (19)	0.0220 (17)	−0.003 (2)
C4	0.0219 (19)	0.046 (3)	0.048 (2)	0.0007 (19)	0.0152 (18)	−0.006 (2)
C5	0.0222 (19)	0.040 (3)	0.041 (2)	0.0043 (19)	0.0099 (18)	−0.001 (2)
C6	0.031 (2)	0.044 (3)	0.059 (3)	0.009 (2)	0.020 (2)	−0.004 (2)
C7	0.026 (2)	0.051 (3)	0.050 (3)	0.001 (2)	0.002 (2)	−0.001 (2)
C8	0.0259 (19)	0.030 (2)	0.0295 (18)	0.0019 (17)	0.0092 (16)	−0.0008 (17)
C9	0.0223 (18)	0.025 (2)	0.0264 (17)	0.0028 (16)	0.0110 (15)	−0.0024 (16)
C10	0.0214 (17)	0.0225 (19)	0.0170 (15)	0.0055 (15)	0.0074 (13)	0.0003 (14)
C11	0.0284 (19)	0.0195 (19)	0.0262 (17)	0.0072 (16)	0.0138 (15)	0.0011 (15)
C12	0.0295 (19)	0.020 (2)	0.0328 (19)	−0.0033 (17)	0.0182 (17)	−0.0036 (16)
C13	0.0237 (17)	0.0172 (17)	0.0238 (16)	0.0019 (15)	0.0121 (14)	0.0024 (15)
C14	0.0228 (17)	0.0236 (19)	0.0287 (18)	0.0016 (16)	0.0136 (15)	0.0042 (16)
C15	0.0264 (19)	0.031 (2)	0.036 (2)	−0.0015 (18)	0.0104 (17)	−0.0014 (18)
C16	0.031 (2)	0.034 (2)	0.047 (2)	0.0059 (19)	0.022 (2)	0.017 (2)
C17	0.039 (2)	0.043 (3)	0.057 (3)	0.005 (2)	0.030 (2)	0.019 (2)
C18	0.074 (5)	0.045 (3)	0.071 (5)	−0.004 (3)	0.060 (4)	−0.006 (4)
C18'	0.074 (5)	0.045 (3)	0.071 (5)	−0.004 (3)	0.060 (4)	−0.006 (4)
C19	0.043 (2)	0.037 (2)	0.048 (3)	0.005 (2)	0.034 (2)	0.003 (2)
C20	0.044 (3)	0.074 (4)	0.049 (3)	0.021 (3)	0.027 (2)	−0.009 (3)
C21	0.035 (3)	0.123 (6)	0.052 (3)	0.021 (3)	0.023 (2)	−0.004 (4)
C22	0.0254 (18)	0.028 (2)	0.0261 (18)	0.0005 (16)	0.0147 (15)	0.0033 (15)
C23	0.035 (2)	0.023 (2)	0.035 (2)	0.0045 (18)	0.0221 (18)	0.0053 (17)
C24	0.034 (2)	0.0180 (18)	0.036 (2)	0.0050 (17)	0.0220 (17)	0.0046 (16)
C25	0.0258 (17)	0.0184 (18)	0.0254 (17)	0.0012 (15)	0.0144 (14)	0.0009 (15)
C26	0.0252 (17)	0.029 (2)	0.0246 (16)	0.0035 (17)	0.0132 (14)	0.0060 (16)
C27	0.0264 (17)	0.0184 (18)	0.0245 (17)	0.0018 (16)	0.0142 (14)	−0.0009 (15)
C28	0.0300 (19)	0.027 (2)	0.0245 (17)	−0.0022 (17)	0.0135 (15)	−0.0049 (16)
C29	0.032 (2)	0.0163 (18)	0.0310 (19)	−0.0024 (16)	0.0189 (17)	−0.0027 (15)
C30	0.028 (2)	0.025 (2)	0.0266 (17)	−0.0041 (16)	0.0152 (16)	−0.0007 (16)
C31	0.036 (2)	0.032 (2)	0.0308 (19)	0.001 (2)	0.0149 (17)	−0.0063 (19)
C32	0.034 (2)	0.040 (3)	0.0271 (18)	−0.006 (2)	0.0146 (16)	−0.0055 (19)
C33	0.039 (2)	0.053 (3)	0.040 (2)	−0.011 (2)	0.022 (2)	−0.006 (2)
C34	0.048 (3)	0.079 (4)	0.038 (2)	−0.025 (3)	0.022 (2)	−0.003 (3)
C35	0.044 (3)	0.077 (4)	0.040 (3)	−0.032 (3)	0.009 (2)	0.009 (3)
C36	0.049 (3)	0.068 (4)	0.071 (4)	−0.024 (3)	0.012 (3)	0.018 (3)
C37	0.059 (4)	0.125 (7)	0.051 (3)	−0.041 (4)	0.016 (3)	0.030 (4)
C38	0.037 (2)	0.053 (3)	0.036 (2)	−0.018 (2)	0.008 (2)	0.008 (2)
C39	0.030 (2)	0.038 (3)	0.0291 (19)	−0.0081 (19)	0.0073 (17)	0.0003 (19)
C40	0.0247 (19)	0.033 (2)	0.0253 (18)	0.0014 (18)	0.0048 (16)	−0.0008 (17)
C41	0.033 (2)	0.030 (2)	0.050 (3)	−0.0028 (19)	0.014 (2)	−0.013 (2)
C42	0.035 (2)	0.042 (3)	0.063 (3)	−0.002 (2)	0.020 (2)	−0.025 (3)
C43	0.0274 (19)	0.034 (2)	0.0240 (17)	0.0042 (18)	0.0086 (15)	−0.0036 (17)
C44	0.0255 (19)	0.043 (3)	0.0225 (17)	0.0096 (19)	0.0070 (15)	0.0010 (18)
C45	0.034 (2)	0.100 (5)	0.029 (2)	0.013 (3)	0.0128 (18)	−0.012 (3)
C46	0.029 (2)	0.043 (3)	0.038 (2)	0.006 (2)	0.0109 (18)	−0.010 (2)
C47	0.031 (2)	0.048 (3)	0.051 (3)	0.011 (2)	0.017 (2)	−0.002 (2)
C48	0.037 (2)	0.049 (3)	0.039 (2)	0.010 (2)	0.019 (2)	0.012 (2)
C49	0.043 (3)	0.048 (3)	0.044 (2)	0.015 (2)	0.027 (2)	0.021 (2)
C50	0.074 (4)	0.038 (3)	0.126 (6)	−0.005 (3)	0.072 (4)	0.003 (3)
C51	0.064 (4)	0.105 (6)	0.070 (4)	0.044 (4)	0.049 (3)	0.058 (4)
C52	0.034 (2)	0.042 (3)	0.0283 (19)	0.012 (2)	0.0177 (17)	0.0119 (18)
C53	0.044 (3)	0.045 (3)	0.046 (3)	0.019 (2)	0.028 (2)	0.023 (2)
C54	0.046 (3)	0.032 (2)	0.044 (2)	0.014 (2)	0.031 (2)	0.012 (2)
C55	0.032 (2)	0.036 (2)	0.0225 (17)	0.0056 (19)	0.0133 (16)	0.0011 (17)
C56	0.034 (2)	0.075 (4)	0.0233 (18)	0.019 (2)	0.0122 (17)	0.006 (2)
C57	0.031 (2)	0.035 (2)	0.0225 (17)	0.0000 (18)	0.0128 (16)	−0.0034 (16)
C58	0.032 (2)	0.043 (2)	0.0237 (18)	−0.0104 (19)	0.0139 (16)	−0.0022 (17)
C59	0.035 (2)	0.034 (2)	0.036 (2)	−0.0038 (19)	0.0217 (18)	−0.0114 (18)
C60	0.034 (2)	0.041 (3)	0.036 (2)	−0.004 (2)	0.0209 (18)	−0.010 (2)
C61	0.119 (9)	0.086 (9)	0.066 (7)	0.030 (7)	0.044 (7)	−0.007 (6)
C63	0.130 (11)	0.126 (12)	0.155 (12)	0.005 (9)	0.067 (9)	−0.010 (9)
C64	0.182 (15)	0.173 (16)	0.178 (15)	0.007 (10)	0.089 (11)	0.008 (10)
C62	0.140 (11)	0.131 (12)	0.113 (10)	0.002 (9)	0.065 (9)	−0.018 (9)
C65	0.135 (12)	0.124 (12)	0.142 (12)	0.000 (9)	0.053 (9)	−0.013 (9)
C66	0.157 (16)	0.163 (14)	0.161 (13)	−0.013 (9)	0.081 (10)	0.006 (10)

### Geometric parameters (Å, °)

**Table:**

O1—C1	1.292 (5)	C33—C34	1.527 (7)
O1—H1	0.8400	C33—H33A	0.9900
O2—C1	1.233 (5)	C33—H33B	0.9900
O3—C18	1.294 (8)	C34—C35	1.528 (10)
O3'—C18'	1.091 (15)	C34—H34A	0.9900
O4—C31	1.220 (6)	C34—H34B	0.9900
O5—C31	1.297 (6)	C35—C36	1.534 (9)
O5—H5	0.8400	C35—C38	1.546 (7)
O6—C48	1.197 (6)	C35—C37	1.548 (8)
O1W—H1W	0.8399	C36—H36A	0.9800
C1—C2	1.519 (5)	C36—H36B	0.9800
C2—C30	1.533 (6)	C36—H36C	0.9800
C2—C9	1.540 (6)	C37—H37A	0.9800
C2—C3	1.562 (5)	C37—H37B	0.9800
C3—C4	1.530 (6)	C37—H37C	0.9800
C3—H3A	0.9900	C38—C39	1.535 (6)
C3—H3B	0.9900	C38—H38A	0.9900
C4—C5	1.521 (7)	C38—H38B	0.9900
C4—H4A	0.9900	C39—C40	1.525 (6)
C4—H4B	0.9900	C39—H39	1.0000
C5—C7	1.531 (6)	C40—C41	1.327 (6)
C5—C6	1.537 (7)	C40—C57	1.524 (6)
C5—C8	1.538 (6)	C41—C42	1.510 (7)
C6—H6A	0.9800	C41—H41	0.9500
C6—H6B	0.9800	C42—C43	1.525 (7)
C6—H6C	0.9800	C42—H42A	0.9900
C7—H7A	0.9800	C42—H42B	0.9900
C7—H7B	0.9800	C43—C55	1.560 (6)
C7—H7C	0.9800	C43—C44	1.572 (6)
C8—C9	1.546 (5)	C43—H43	1.0000
C8—H8A	0.9900	C44—C46	1.541 (6)
C8—H8B	0.9900	C44—C52	1.540 (7)
C9—C10	1.528 (5)	C44—C45	1.556 (6)
C9—H9	1.0000	C45—H45A	0.9800
C10—C11	1.325 (6)	C45—H45B	0.9800
C10—C27	1.532 (5)	C45—H45C	0.9800
C11—C12	1.503 (5)	C46—C47	1.529 (7)
C11—H11	0.9500	C46—H46A	0.9900
C12—C13	1.537 (5)	C46—H46B	0.9900
C12—H12A	0.9900	C47—C48	1.490 (8)
C12—H12B	0.9900	C47—H47A	0.9900
C13—C25	1.550 (5)	C47—H47B	0.9900
C13—C14	1.566 (5)	C48—C49	1.533 (7)
C13—H13	1.0000	C49—C50	1.504 (9)
C14—C15	1.530 (6)	C49—C51	1.545 (7)
C14—C22	1.540 (6)	C49—C52	1.571 (6)
C14—C16	1.545 (6)	C50—H50A	0.9800
C15—H15A	0.9800	C50—H50B	0.9800
C15—H15B	0.9800	C50—H50C	0.9800
C15—H15C	0.9800	C51—H51A	0.9800
C16—C17	1.535 (6)	C51—H51B	0.9800
C16—H16A	0.9900	C51—H51C	0.9800
C16—H16B	0.9900	C52—C53	1.515 (6)
C17—C18	1.471 (9)	C52—H52	1.0000
C17—C18'	1.481 (12)	C53—C54	1.532 (6)
C17—H17A	0.9900	C53—H53A	0.9900
C17—H17B	0.9900	C53—H53B	0.9900
C18—C19	1.495 (8)	C54—C55	1.538 (7)
C18'—C19	1.533 (12)	C54—H54A	0.9900
C19—C20	1.535 (7)	C54—H54B	0.9900
C19—C21	1.550 (8)	C55—C56	1.537 (5)
C19—C22	1.566 (6)	C55—C57	1.596 (5)
C20—H20A	0.9800	C56—H56A	0.9800
C20—H20B	0.9800	C56—H56B	0.9800
C20—H20C	0.9800	C56—H56C	0.9800
C21—H21A	0.9800	C57—C59	1.546 (6)
C21—H21B	0.9800	C57—C58	1.546 (5)
C21—H21C	0.9800	C58—H58A	0.9800
C22—C23	1.534 (5)	C58—H58B	0.9800
C22—H22	1.0000	C58—H58C	0.9800
C23—C24	1.530 (6)	C59—C60	1.533 (6)
C23—H23A	0.9900	C59—H59A	0.9900
C23—H23B	0.9900	C59—H59B	0.9900
C24—C25	1.540 (5)	C60—H60A	0.9900
C24—H24A	0.9900	C60—H60B	0.9900
C24—H24B	0.9900	C61—C62	1.504 (10)
C25—C26	1.548 (5)	C61—H61A	0.9800
C25—C27	1.592 (5)	C61—H61B	0.9800
C26—H26A	0.9800	C61—H61C	0.9800
C26—H26B	0.9800	C63—C62	1.540 (10)
C26—H26C	0.9800	C63—C64	1.560 (11)
C27—C29	1.540 (5)	C63—H63A	0.9900
C27—C28	1.560 (5)	C63—H63B	0.9900
C28—H28A	0.9800	C64—C65	1.548 (11)
C28—H28B	0.9800	C64—H64A	0.9900
C28—H28C	0.9800	C64—H64B	0.9900
C29—C30	1.525 (5)	C62—H62A	0.9900
C29—H29A	0.9900	C62—H62B	0.9900
C29—H29B	0.9900	C65—C66	1.527 (11)
C30—H30A	0.9900	C65—H65A	0.9900
C30—H30B	0.9900	C65—H65B	0.9900
C31—C32	1.519 (6)	C66—H66A	0.9800
C32—C60	1.530 (7)	C66—H66B	0.9800
C32—C39	1.537 (7)	C66—H66C	0.9800
C32—C33	1.548 (6)		
			
C1—O1—H1	109.5	C34—C33—H33B	109.1
C31—O5—H5	109.5	C32—C33—H33B	109.1
O2—C1—O1	122.6 (3)	H33A—C33—H33B	107.8
O2—C1—C2	121.6 (4)	C33—C34—C35	113.1 (5)
O1—C1—C2	115.7 (4)	C33—C34—H34A	109.0
C1—C2—C30	111.6 (3)	C35—C34—H34A	109.0
C1—C2—C9	109.9 (3)	C33—C34—H34B	109.0
C30—C2—C9	110.1 (3)	C35—C34—H34B	109.0
C1—C2—C3	103.1 (3)	H34A—C34—H34B	107.8
C30—C2—C3	110.3 (3)	C34—C35—C36	111.4 (5)
C9—C2—C3	111.8 (3)	C34—C35—C38	108.0 (5)
C4—C3—C2	112.5 (3)	C36—C35—C38	111.5 (6)
C4—C3—H3A	109.1	C34—C35—C37	107.0 (6)
C2—C3—H3A	109.1	C36—C35—C37	109.9 (5)
C4—C3—H3B	109.1	C38—C35—C37	108.9 (4)
C2—C3—H3B	109.1	C35—C36—H36A	109.5
H3A—C3—H3B	107.8	C35—C36—H36B	109.5
C5—C4—C3	112.7 (4)	H36A—C36—H36B	109.5
C5—C4—H4A	109.1	C35—C36—H36C	109.5
C3—C4—H4A	109.1	H36A—C36—H36C	109.5
C5—C4—H4B	109.1	H36B—C36—H36C	109.5
C3—C4—H4B	109.1	C35—C37—H37A	109.5
H4A—C4—H4B	107.8	C35—C37—H37B	109.5
C4—C5—C7	109.1 (4)	H37A—C37—H37B	109.5
C4—C5—C6	110.7 (4)	C35—C37—H37C	109.5
C7—C5—C6	108.8 (4)	H37A—C37—H37C	109.5
C4—C5—C8	108.7 (4)	H37B—C37—H37C	109.5
C7—C5—C8	109.3 (4)	C39—C38—C35	113.5 (4)
C6—C5—C8	110.2 (4)	C39—C38—H38A	108.9
C5—C6—H6A	109.5	C35—C38—H38A	108.9
C5—C6—H6B	109.5	C39—C38—H38B	108.9
H6A—C6—H6B	109.5	C35—C38—H38B	108.9
C5—C6—H6C	109.5	H38A—C38—H38B	107.7
H6A—C6—H6C	109.5	C40—C39—C38	113.5 (4)
H6B—C6—H6C	109.5	C40—C39—C32	111.9 (4)
C5—C7—H7A	109.5	C38—C39—C32	111.0 (4)
C5—C7—H7B	109.5	C40—C39—H39	106.7
H7A—C7—H7B	109.5	C38—C39—H39	106.7
C5—C7—H7C	109.5	C32—C39—H39	106.7
H7A—C7—H7C	109.5	C41—C40—C57	120.7 (4)
H7B—C7—H7C	109.5	C41—C40—C39	119.1 (4)
C5—C8—C9	114.5 (3)	C57—C40—C39	120.2 (4)
C5—C8—H8A	108.6	C40—C41—C42	126.0 (4)
C9—C8—H8A	108.6	C40—C41—H41	117.0
C5—C8—H8B	108.6	C42—C41—H41	117.0
C9—C8—H8B	108.6	C41—C42—C43	113.7 (4)
H8A—C8—H8B	107.6	C41—C42—H42A	108.8
C10—C9—C2	111.4 (3)	C43—C42—H42A	108.8
C10—C9—C8	112.4 (3)	C41—C42—H42B	108.8
C2—C9—C8	110.9 (3)	C43—C42—H42B	108.8
C10—C9—H9	107.3	H42A—C42—H42B	107.7
C2—C9—H9	107.3	C42—C43—C55	109.9 (3)
C8—C9—H9	107.3	C42—C43—C44	114.2 (4)
C11—C10—C9	119.3 (3)	C55—C43—C44	116.9 (4)
C11—C10—C27	119.9 (3)	C42—C43—H43	104.8
C9—C10—C27	120.7 (3)	C55—C43—H43	104.8
C10—C11—C12	126.2 (3)	C44—C43—H43	104.8
C10—C11—H11	116.9	C46—C44—C52	108.6 (3)
C12—C11—H11	116.9	C46—C44—C45	108.1 (4)
C11—C12—C13	114.1 (3)	C52—C44—C45	112.8 (4)
C11—C12—H12A	108.7	C46—C44—C43	107.2 (4)
C13—C12—H12A	108.7	C52—C44—C43	106.5 (3)
C11—C12—H12B	108.7	C45—C44—C43	113.5 (4)
C13—C12—H12B	108.7	C44—C45—H45A	109.5
H12A—C12—H12B	107.6	C44—C45—H45B	109.5
C12—C13—C25	110.4 (3)	H45A—C45—H45B	109.5
C12—C13—C14	113.9 (3)	C44—C45—H45C	109.5
C25—C13—C14	117.4 (3)	H45A—C45—H45C	109.5
C12—C13—H13	104.5	H45B—C45—H45C	109.5
C25—C13—H13	104.5	C47—C46—C44	112.4 (4)
C14—C13—H13	104.5	C47—C46—H46A	109.1
C15—C14—C22	112.8 (3)	C44—C46—H46A	109.1
C15—C14—C16	108.8 (4)	C47—C46—H46B	109.1
C22—C14—C16	106.5 (3)	C44—C46—H46B	109.1
C15—C14—C13	114.2 (3)	H46A—C46—H46B	107.9
C22—C14—C13	107.6 (3)	C48—C47—C46	112.2 (4)
C16—C14—C13	106.5 (3)	C48—C47—H47A	109.2
C14—C15—H15A	109.5	C46—C47—H47A	109.2
C14—C15—H15B	109.5	C48—C47—H47B	109.2
H15A—C15—H15B	109.5	C46—C47—H47B	109.2
C14—C15—H15C	109.5	H47A—C47—H47B	107.9
H15A—C15—H15C	109.5	O6—C48—C47	122.0 (5)
H15B—C15—H15C	109.5	O6—C48—C49	121.3 (5)
C17—C16—C14	112.9 (3)	C47—C48—C49	116.7 (4)
C17—C16—H16A	109.0	C50—C49—C48	108.4 (5)
C14—C16—H16A	109.0	C50—C49—C51	107.9 (5)
C17—C16—H16B	109.0	C48—C49—C51	107.9 (4)
C14—C16—H16B	109.0	C50—C49—C52	110.8 (4)
H16A—C16—H16B	107.8	C48—C49—C52	107.3 (4)
C18—C17—C18'	25.9 (4)	C51—C49—C52	114.3 (4)
C18—C17—C16	114.8 (4)	C49—C50—H50A	109.5
C18'—C17—C16	110.6 (5)	C49—C50—H50B	109.5
C18—C17—H17A	108.6	H50A—C50—H50B	109.5
C18'—C17—H17A	87.1	C49—C50—H50C	109.5
C16—C17—H17A	108.6	H50A—C50—H50C	109.5
C18—C17—H17B	108.6	H50B—C50—H50C	109.5
C18'—C17—H17B	130.7	C49—C51—H51A	109.5
C16—C17—H17B	108.6	C49—C51—H51B	109.5
H17A—C17—H17B	107.5	H51A—C51—H51B	109.5
O3—C18—C17	116.7 (6)	C49—C51—H51C	109.5
O3—C18—C19	117.5 (6)	H51A—C51—H51C	109.5
C17—C18—C19	125.9 (5)	H51B—C51—H51C	109.5
O3'—C18'—C17	115.5 (10)	C53—C52—C44	111.0 (4)
O3'—C18'—C19	122.2 (11)	C53—C52—C49	113.3 (4)
C17—C18'—C19	122.3 (9)	C44—C52—C49	118.8 (4)
C18—C19—C18'	25.2 (3)	C53—C52—H52	104.0
C18—C19—C20	109.4 (4)	C44—C52—H52	104.0
C18'—C19—C20	88.1 (5)	C49—C52—H52	104.0
C18—C19—C21	106.9 (5)	C52—C53—C54	110.1 (4)
C18'—C19—C21	127.8 (5)	C52—C53—H53A	109.6
C20—C19—C21	106.9 (5)	C54—C53—H53A	109.6
C18—C19—C22	110.8 (4)	C52—C53—H53B	109.6
C18'—C19—C22	107.1 (5)	C54—C53—H53B	109.6
C20—C19—C22	108.9 (4)	H53A—C53—H53B	108.2
C21—C19—C22	113.9 (4)	C53—C54—C55	114.6 (4)
C19—C20—H20A	109.5	C53—C54—H54A	108.6
C19—C20—H20B	109.5	C55—C54—H54A	108.6
H20A—C20—H20B	109.5	C53—C54—H54B	108.6
C19—C20—H20C	109.5	C55—C54—H54B	108.6
H20A—C20—H20C	109.5	H54A—C54—H54B	107.6
H20B—C20—H20C	109.5	C56—C55—C54	108.3 (4)
C19—C21—H21A	109.5	C56—C55—C43	110.6 (3)
C19—C21—H21B	109.5	C54—C55—C43	110.5 (3)
H21A—C21—H21B	109.5	C56—C55—C57	110.0 (3)
C19—C21—H21C	109.5	C54—C55—C57	109.9 (3)
H21A—C21—H21C	109.5	C43—C55—C57	107.5 (3)
H21B—C21—H21C	109.5	C55—C56—H56A	109.5
C23—C22—C14	111.7 (3)	C55—C56—H56B	109.5
C23—C22—C19	112.0 (3)	H56A—C56—H56B	109.5
C14—C22—C19	115.8 (3)	C55—C56—H56C	109.5
C23—C22—H22	105.5	H56A—C56—H56C	109.5
C14—C22—H22	105.5	H56B—C56—H56C	109.5
C19—C22—H22	105.5	C40—C57—C59	112.7 (3)
C24—C23—C22	109.6 (3)	C40—C57—C58	106.8 (3)
C24—C23—H23A	109.7	C59—C57—C58	106.8 (3)
C22—C23—H23A	109.7	C40—C57—C55	109.1 (3)
C24—C23—H23B	109.7	C59—C57—C55	109.1 (3)
C22—C23—H23B	109.7	C58—C57—C55	112.3 (3)
H23A—C23—H23B	108.2	C57—C58—H58A	109.5
C23—C24—C25	113.5 (3)	C57—C58—H58B	109.5
C23—C24—H24A	108.9	H58A—C58—H58B	109.5
C25—C24—H24A	108.9	C57—C58—H58C	109.5
C23—C24—H24B	108.9	H58A—C58—H58C	109.5
C25—C24—H24B	108.9	H58B—C58—H58C	109.5
H24A—C24—H24B	107.7	C60—C59—C57	114.6 (4)
C24—C25—C26	108.8 (3)	C60—C59—H59A	108.6
C24—C25—C13	110.4 (3)	C57—C59—H59A	108.6
C26—C25—C13	110.4 (3)	C60—C59—H59B	108.6
C24—C25—C27	109.8 (3)	C57—C59—H59B	108.6
C26—C25—C27	110.2 (3)	H59A—C59—H59B	107.6
C13—C25—C27	107.3 (3)	C32—C60—C59	112.5 (3)
C25—C26—H26A	109.5	C32—C60—H60A	109.1
C25—C26—H26B	109.5	C59—C60—H60A	109.1
H26A—C26—H26B	109.5	C32—C60—H60B	109.1
C25—C26—H26C	109.5	C59—C60—H60B	109.1
H26A—C26—H26C	109.5	H60A—C60—H60B	107.8
H26B—C26—H26C	109.5	C62—C61—H61A	109.5
C10—C27—C29	113.0 (3)	C62—C61—H61B	109.5
C10—C27—C28	106.9 (3)	H61A—C61—H61B	109.5
C29—C27—C28	107.3 (3)	C62—C61—H61C	109.5
C10—C27—C25	108.3 (3)	H61A—C61—H61C	109.5
C29—C27—C25	108.9 (3)	H61B—C61—H61C	109.5
C28—C27—C25	112.6 (3)	C62—C63—C64	107.0 (9)
C27—C28—H28A	109.5	C62—C63—H63A	110.3
C27—C28—H28B	109.5	C64—C63—H63A	110.3
H28A—C28—H28B	109.5	C62—C63—H63B	110.3
C27—C28—H28C	109.5	C64—C63—H63B	110.3
H28A—C28—H28C	109.5	H63A—C63—H63B	108.6
H28B—C28—H28C	109.5	C65—C64—C63	106.6 (9)
C30—C29—C27	114.6 (3)	C65—C64—H64A	110.4
C30—C29—H29A	108.6	C63—C64—H64A	110.4
C27—C29—H29A	108.6	C65—C64—H64B	110.4
C30—C29—H29B	108.6	C63—C64—H64B	110.4
C27—C29—H29B	108.6	H64A—C64—H64B	108.6
H29A—C29—H29B	107.6	C61—C62—C63	110.8 (10)
C29—C30—C2	111.9 (3)	C61—C62—H62A	109.5
C29—C30—H30A	109.2	C63—C62—H62A	109.5
C2—C30—H30A	109.2	C61—C62—H62B	109.5
C29—C30—H30B	109.2	C63—C62—H62B	109.5
C2—C30—H30B	109.2	H62A—C62—H62B	108.1
H30A—C30—H30B	107.9	C66—C65—C64	109.2 (10)
O4—C31—O5	122.0 (4)	C66—C65—H65A	109.8
O4—C31—C32	120.9 (4)	C64—C65—H65A	109.8
O5—C31—C32	117.0 (4)	C66—C65—H65B	109.8
C31—C32—C60	111.9 (4)	C64—C65—H65B	109.8
C31—C32—C39	108.6 (3)	H65A—C65—H65B	108.3
C60—C32—C39	109.4 (3)	C65—C66—H66A	109.5
C31—C32—C33	103.0 (3)	C65—C66—H66B	109.5
C60—C32—C33	111.5 (4)	H66A—C66—H66B	109.5
C39—C32—C33	112.4 (4)	C65—C66—H66C	109.5
C34—C33—C32	112.6 (4)	H66A—C66—H66C	109.5
C34—C33—H33A	109.1	H66B—C66—H66C	109.5
C32—C33—H33A	109.1		
			
O2—C1—C2—C30	156.5 (4)	C13—C25—C27—C28	57.6 (4)
O1—C1—C2—C30	−27.4 (5)	C10—C27—C29—C30	37.2 (4)
O2—C1—C2—C9	34.1 (5)	C28—C27—C29—C30	−80.3 (4)
O1—C1—C2—C9	−149.9 (3)	C25—C27—C29—C30	157.6 (3)
O2—C1—C2—C3	−85.2 (5)	C27—C29—C30—C2	−53.9 (4)
O1—C1—C2—C3	90.9 (4)	C1—C2—C30—C29	−61.1 (4)
C1—C2—C3—C4	169.1 (4)	C9—C2—C30—C29	61.2 (4)
C30—C2—C3—C4	−71.6 (5)	C3—C2—C30—C29	−175.0 (3)
C9—C2—C3—C4	51.2 (5)	O4—C31—C32—C60	152.6 (5)
C2—C3—C4—C5	−55.4 (5)	O5—C31—C32—C60	−31.2 (6)
C3—C4—C5—C7	175.2 (4)	O4—C31—C32—C39	31.8 (6)
C3—C4—C5—C6	−65.1 (5)	O5—C31—C32—C39	−152.0 (4)
C3—C4—C5—C8	56.1 (5)	O4—C31—C32—C33	−87.5 (6)
C4—C5—C8—C9	−56.1 (5)	O5—C31—C32—C33	88.7 (5)
C7—C5—C8—C9	−175.0 (4)	C31—C32—C33—C34	166.4 (5)
C6—C5—C8—C9	65.3 (5)	C60—C32—C33—C34	−73.4 (6)
C1—C2—C9—C10	70.8 (4)	C39—C32—C33—C34	49.8 (6)
C30—C2—C9—C10	−52.5 (4)	C32—C33—C34—C35	−54.0 (6)
C3—C2—C9—C10	−175.4 (3)	C33—C34—C35—C36	−66.5 (6)
C1—C2—C9—C8	−163.1 (3)	C33—C34—C35—C38	56.2 (6)
C30—C2—C9—C8	73.5 (4)	C33—C34—C35—C37	173.4 (4)
C3—C2—C9—C8	−49.3 (4)	C34—C35—C38—C39	−57.6 (6)
C5—C8—C9—C10	179.2 (4)	C36—C35—C38—C39	65.1 (7)
C5—C8—C9—C2	53.7 (5)	C37—C35—C38—C39	−173.4 (6)
C2—C9—C10—C11	−144.3 (3)	C35—C38—C39—C40	−177.6 (5)
C8—C9—C10—C11	90.4 (4)	C35—C38—C39—C32	55.4 (6)
C2—C9—C10—C27	39.6 (4)	C31—C32—C39—C40	68.9 (4)
C8—C9—C10—C27	−85.6 (4)	C60—C32—C39—C40	−53.4 (4)
C9—C10—C11—C12	−179.0 (3)	C33—C32—C39—C40	−177.8 (3)
C27—C10—C11—C12	−2.9 (6)	C31—C32—C39—C38	−163.2 (4)
C10—C11—C12—C13	2.9 (5)	C60—C32—C39—C38	74.5 (4)
C11—C12—C13—C25	−33.3 (4)	C33—C32—C39—C38	−49.9 (5)
C11—C12—C13—C14	−167.9 (3)	C38—C39—C40—C41	93.5 (5)
C12—C13—C14—C15	54.9 (4)	C32—C39—C40—C41	−139.9 (4)
C25—C13—C14—C15	−76.4 (4)	C38—C39—C40—C57	−84.2 (5)
C12—C13—C14—C22	−179.0 (3)	C32—C39—C40—C57	42.3 (5)
C25—C13—C14—C22	49.6 (4)	C57—C40—C41—C42	−1.8 (7)
C12—C13—C14—C16	−65.2 (4)	C39—C40—C41—C42	−179.5 (4)
C25—C13—C14—C16	163.5 (3)	C40—C41—C42—C43	6.7 (7)
C15—C14—C16—C17	62.0 (5)	C41—C42—C43—C55	−37.9 (5)
C22—C14—C16—C17	−59.8 (5)	C41—C42—C43—C44	−171.6 (4)
C13—C14—C16—C17	−174.4 (4)	C42—C43—C44—C46	−61.7 (5)
C14—C16—C17—C18	24.3 (6)	C55—C43—C44—C46	168.0 (3)
C14—C16—C17—C18'	52.0 (6)	C42—C43—C44—C52	−177.7 (4)
C18'—C17—C18—O3	111.9 (11)	C55—C43—C44—C52	52.0 (4)
C16—C17—C18—O3	−161.8 (5)	C42—C43—C44—C45	57.6 (6)
C18'—C17—C18—C19	−69.0 (11)	C55—C43—C44—C45	−72.7 (5)
C16—C17—C18—C19	17.3 (5)	C52—C44—C46—C47	−52.4 (5)
C18—C17—C18'—O3'	−119.0 (10)	C45—C44—C46—C47	70.2 (5)
C16—C17—C18'—O3'	136.4 (6)	C43—C44—C46—C47	−167.1 (4)
C18—C17—C18'—C19	60.8 (10)	C44—C46—C47—C48	56.1 (5)
C16—C17—C18'—C19	−43.8 (6)	C46—C47—C48—O6	123.7 (6)
O3—C18—C19—C18'	−113.2 (11)	C46—C47—C48—C49	−54.6 (6)
C17—C18—C19—C18'	67.7 (11)	O6—C48—C49—C50	−11.4 (7)
O3—C18—C19—C20	−79.3 (4)	C47—C48—C49—C50	166.9 (5)
C17—C18—C19—C20	101.6 (4)	O6—C48—C49—C51	105.2 (6)
O3—C18—C19—C21	36.0 (4)	C47—C48—C49—C51	−76.5 (6)
C17—C18—C19—C21	−143.1 (4)	O6—C48—C49—C52	−131.1 (5)
O3—C18—C19—C22	160.6 (4)	C47—C48—C49—C52	47.1 (5)
C17—C18—C19—C22	−18.5 (4)	C46—C44—C52—C53	−175.7 (4)
O3'—C18'—C19—C18	118.0 (10)	C45—C44—C52—C53	64.5 (5)
C17—C18'—C19—C18	−61.7 (10)	C43—C44—C52—C53	−60.6 (4)
O3'—C18'—C19—C20	−30.2 (6)	C46—C44—C52—C49	50.3 (5)
C17—C18'—C19—C20	150.0 (5)	C45—C44—C52—C49	−69.5 (5)
O3'—C18'—C19—C21	79.8 (8)	C43—C44—C52—C49	165.4 (3)
C17—C18'—C19—C21	−100.0 (7)	C50—C49—C52—C53	62.5 (5)
O3'—C18'—C19—C22	−139.5 (6)	C48—C49—C52—C53	−179.3 (4)
C17—C18'—C19—C22	40.8 (6)	C51—C49—C52—C53	−59.7 (6)
C15—C14—C22—C23	69.9 (4)	C50—C49—C52—C44	−164.6 (4)
C16—C14—C22—C23	−170.8 (3)	C48—C49—C52—C44	−46.4 (5)
C13—C14—C22—C23	−56.9 (4)	C51—C49—C52—C44	73.2 (6)
C15—C14—C22—C19	−59.8 (4)	C44—C52—C53—C54	64.0 (5)
C16—C14—C22—C19	59.4 (4)	C49—C52—C53—C54	−159.4 (4)
C13—C14—C22—C19	173.3 (3)	C52—C53—C54—C55	−55.3 (5)
C18—C19—C22—C23	−151.5 (3)	C53—C54—C55—C56	−76.9 (5)
C18'—C19—C22—C23	−177.9 (5)	C53—C54—C55—C43	44.4 (5)
C20—C19—C22—C23	88.1 (5)	C53—C54—C55—C57	162.9 (4)
C21—C19—C22—C23	−31.0 (6)	C42—C43—C55—C56	−56.7 (5)
C18—C19—C22—C14	−21.9 (4)	C44—C43—C55—C56	75.7 (5)
C18'—C19—C22—C14	−48.2 (5)	C42—C43—C55—C54	−176.6 (4)
C20—C19—C22—C14	−142.2 (4)	C44—C43—C55—C54	−44.3 (4)
C21—C19—C22—C14	98.7 (5)	C42—C43—C55—C57	63.4 (4)
C14—C22—C23—C24	62.8 (4)	C44—C43—C55—C57	−164.2 (3)
C19—C22—C23—C24	−165.5 (3)	C41—C40—C57—C59	149.0 (4)
C22—C23—C24—C25	−57.7 (4)	C39—C40—C57—C59	−33.3 (5)
C23—C24—C25—C26	−72.9 (4)	C41—C40—C57—C58	−94.0 (5)
C23—C24—C25—C13	48.3 (4)	C39—C40—C57—C58	83.7 (4)
C23—C24—C25—C27	166.4 (3)	C41—C40—C57—C55	27.7 (5)
C12—C13—C25—C24	−178.4 (3)	C39—C40—C57—C55	−154.6 (4)
C14—C13—C25—C24	−45.5 (4)	C56—C55—C57—C40	63.1 (5)
C12—C13—C25—C26	−58.2 (4)	C54—C55—C57—C40	−177.7 (3)
C14—C13—C25—C26	74.7 (4)	C43—C55—C57—C40	−57.4 (4)
C12—C13—C25—C27	61.9 (4)	C56—C55—C57—C59	−60.3 (5)
C14—C13—C25—C27	−165.2 (3)	C54—C55—C57—C59	58.9 (4)
C11—C10—C27—C29	152.6 (3)	C43—C55—C57—C59	179.2 (3)
C9—C10—C27—C29	−31.4 (4)	C56—C55—C57—C58	−178.6 (4)
C11—C10—C27—C28	−89.6 (4)	C54—C55—C57—C58	−59.4 (5)
C9—C10—C27—C28	86.4 (4)	C43—C55—C57—C58	60.9 (4)
C11—C10—C27—C25	31.9 (4)	C40—C57—C59—C60	37.2 (5)
C9—C10—C27—C25	−152.1 (3)	C58—C57—C59—C60	−79.8 (4)
C24—C25—C27—C10	179.6 (3)	C55—C57—C59—C60	158.5 (3)
C26—C25—C27—C10	59.8 (4)	C31—C32—C60—C59	−60.2 (5)
C13—C25—C27—C10	−60.4 (3)	C39—C32—C60—C59	60.2 (5)
C24—C25—C27—C29	56.4 (4)	C33—C32—C60—C59	−174.9 (4)
C26—C25—C27—C29	−63.4 (4)	C57—C59—C60—C32	−52.8 (5)
C13—C25—C27—C29	176.4 (3)	C62—C63—C64—C65	−159 (2)
C24—C25—C27—C28	−62.4 (4)	C64—C63—C62—C61	−73 (2)
C26—C25—C27—C28	177.8 (3)	C63—C64—C65—C66	180 (2)

### Hydrogen-bond geometry (Å, °)

**Table:**

*D*—H···*A*	*D*—H	H···*A*	*D*···*A*	*D*—H···*A*
O1—H1···O4	0.84	1.71	2.545 (4)	171
O5—H5···O2	0.84	1.83	2.637 (4)	160
O1w—H1w···O3	0.84	2.20	3.03 (2)	169
